# An evaluation of the Royal College of Psychiatrists' “Psych Star” scheme

**DOI:** 10.1192/bjo.2021.410

**Published:** 2021-06-18

**Authors:** India Lunn, Declan Hyland

**Affiliations:** 1 Medical undergraduate, University of Sheffield; 2 Clock View Hospital, Liverpool, Mersey Care NHS Foundation Trust

## Abstract

**Aims:**

In 2019, the Royal College of Psychiatrists (RCPsych) launched the “Psych Star” scheme for medical students with an interest in psychiatry. The one-year scheme provides Psych Stars with a matched mentor, free registration at the RCPsych's International Congress, financial support for psychiatry-related activities, journal subscriptions and access to two online learning platforms. This project aimed to evaluate the effectiveness of the scheme in supporting Psych Stars to explore their interest in psychiatry and in promoting psychiatry as a career choice, through use of a survey for both Psych Stars and mentors.

**Method:**

Surveys were sent to all Psych Stars and mentors from the first cohort of the scheme. The mentor and student surveys contained a mixture of ranking, multiple choice, closed-ended and open-ended questions. Questions examined: clarity of the scheme's aims and objectives; benefits of each aspect of the scheme; impact of the scheme on application to Core Training; benefits and barriers to successful mentorship; adequacy of mentor support from the RCPsych and suggestions to improve the scheme.

**Result:**

Six Psych Stars and nine mentors completed the respective surveys. All Psych Stars stated the scheme was enjoyable. Five Psych Stars were more likely to apply for Core Training because of the scheme. All Psych Stars identified the most beneficial aspect of the scheme being the funded place at the RCPsych International Congress. Other aspects highly ranked included: funding to explore areas of psychiatry of interest and the opportunity to be an ambassador for psychiatry. All Psych Stars found the mentorship useful.

Mentors supported Psych Stars by providing career advice, suggesting relevant conferences to attend and assisting Psych Stars make decisions on how to use their allocated funding. Barriers to mentorship that were identified included: geographical separation, limitations related to the COVID-19 pandemic and lack of time. For mentors, areas for improvement included clearer aims and objectives and more support from the RCPsych.

Both mentors and Psych Stars suggested forming a network of Psych Stars and mentors would be useful to share ideas and experiences.

**Conclusion:**

This evaluation shows that the Psych Star scheme successfully supports Psych Stars to explore their interest in psychiatry, and promotes psychiatry as a career choice. This survey has been helpful in identifying what aspects of the scheme are particularly attractive, and also, importantly, how the Psych Stars scheme can be improved for future cohorts. The survey will be delivered to all future annual cohorts of Psych Stars and mentors.

